# Travis County Medical Association

**Published:** 1887-07

**Authors:** 


### TRAVIS COUNTY MEDICAL ASSOCIATION.

At the last regular meeting of Travis County Medical Association, held in Austin, July 14. Dr. A. N. Denton, late Superintendent State Lunatic Asylum, was elected President, Dr. T. J. Tyner, *the* Oculist, Vice-President, and Dr. Florence E. Collins, re-elected Secretary and Treasurer.

